# Typical Cases of Malarial Inflammation of the Middle Ear

**Published:** 1882-05

**Authors:** 


MEETING OF MAY 1.

Dr. F. C. Hotz reported some typical cases of malarial inflammation of the middle ear. His treatment consisted in giving from three to five grains of quinine four or five times a day, and the cure was complete in a week, and the hearing restored to its primitive condition.

Dr. J. H. Hollister had noticed, as a secondary result of malarial fever, that the secretory membranes were all liable to be affected, and it was a matter of importance to him to see that Dr. Hotz, in his paper, had specialized that morbid action in its relations to the ear.

Asked if the disease was neuralgia, Dr. Hotz answered that it was rather inflammatory and constitutional, and that the impairment of hearing was greater than in ordinary local inflammations.

Dr. Clark remarked that nine-tenths of the cases of pain and buzzing in the ears were referred by the patients to a previous use of quinine, and he believed in such a connection between the two; he knew of some persons who were unable to take that medicine, even in small doses, on account of the buzzing occasioned thereby.

Dr. Rosa Engert related a case of malarial fever in the course of which acute otitis took place, followed by a free discharge of pus. The patient was kept on quinine, two or three grains at short intervals, and made a good recovery, the ear healing in a short time.

Dr. W. T. Montgomery had seen two cases which he classed as malarial otitis, although not very typical in character. A free discharge of pus from the ear was taking place when he saw them. Hot water, astringents, local blood-letting and the various means used in such cases failed to give any relief, and seemed to aggravate the disease. After a few days he gave large doses of quinine, which brought speedy recovery. The treatment in these cases had been the only means of furnishing a diagnosis.

A case resembling the above was reported by Dr. Gardner. A lady who had had malarial fever years ago had subsequently suppurative otitis, with inflammation of the mastoid cells. The disease stopped in her right ear, but in the left it remained stationary. He was called to see the patient after she had had a chill, and he gave three grains of quinine four times a day, which brought on recovery. Dover's powders were also given. Three months later another attack of chills and fever set in, for which he gave quinine, followed with speedy recovery.

Dr. S. J. Jones said we were all well aware of the complications which may accompany malarial fever. He had noticed that the preceding cases were chronic, hence liable to be aggravated by various causes. He had yet to see his first case of malarial otitis. In most cases, he thought, there had been exposition, and their various symptoms were complications and aggravation from malarial irritation. He should look at the related cases as such. We knew very well the beneficial effects of quinine under the circumstances, but any tonic would also give similar results. The periodicity of the attack was not a sufficient proof. He had seen permanent impairment of hearing follow the use of quinine. As to the cases of malarial otitis related, he believed that malarial poisoning had much to do with them, but he would not class them as malarial, except as complications.

Dr. E. Ingals said that it was an old observation that quinine affected the ear markedly, impairing the hearing when given in large doses. The practice had been confined to a malarial district, but he was so convinced of the ill effects of quinine that he always gave it in rather small doses, and never gave it in cases where the hearing was in any way affected.

Dr. Hotz, reviewing the criticisms passed on his reports, said that he certainly did not mean to state that any case of otitis relieved by the administration of quinine was malarial. In almost all cases of chronic catarrhal inflammation he would agree with Dr. Jones; but as to the typical cases, their existence could not be denied. The fact that under ordinary treatment these cases had gone from bad to worse, while the antiperiodic use of quinine brought immediate relief after a case of this nature had extended over months, and healed in a week with quinine, was sufficient evidence to separate it from ordinary catarrhal inflammation. Those cases *do not* get along as well with other tonics, nor did he consider quinine a tonic in such doses and in such cases.

There were cases of inflammation of the ear, purely local, without constitutional disturbances, which he had seen heal without treatment. He had also seen cases in which syringing had been kept up a long while, get better after stopping all treatment.

INSANITY FROM TRAUMATISM.

Dr. William P. Verity, ex-interne of the Cook County Hospital, related a case of this nature which had some medico-legal relations, as the patient committed suicide. In 500 cases of insanity observed by Dr. Kiernan, 49 were classed as traumatic. In these dull pain, fainting, delirium, immediate or subsequent, had occasionally taken place. There had been a tendency to brain congestion, noises in the ear, and in a few cases changes in the pupils and in the smell. There had been outbursts of temper, and, in fourteen cases, attempts at suicide. The prognosis was unfavorable. Certain psychoses were liable to form, the majority of which ended in progressive paresis. Heredity had very little to do with it. Injuries to the brain before the age of forty were more liable to give rise to such diseases than when taking place later. Some cases had well marked delusions.

The case which came under the observation of Dr. Verity was that of a man fifty years old, who had previously enjoyed good health. He was industrious, drank beer moderately, and was careful of his personal appearance. On December 3, 1878, he was thrown on a truck by an engine, and his head hit on one side. He afterward felt pain in that part of his head; he became disinclined to work; his sexual appetite, hitherto moderate, increased, and he became intemperate. He had tinnitus aurium, and became loquacious. December 24, 1881, he struck his wife, and some people tied him down for the night. The next morning, the influence of liquor having passed away, he still menaced to kill his wife. December 26 he hung himself and died. The autopsy showed extensive adhesions of the cerebral meninges, but the brain substance appeared normal. Dr. Verity pronounced the case one of traumatic insanity.

Dr. Kiernan agreed with the author of the paper in his diagnosis, but he did not believe the title quite fitted to represent that class of mental diseases. He was convinced of the fact that very slight injuries had a great bearing on the types of insanity subsequently developed in such cases. Lesions were not always discernible, though probably always present.

Dr. E. Ingals asked whether suicide was not a definite proof of insanity in every case.

Dr. Kiernan was persuaded that he had met sane suicides.

The President related a case of traumatic insanity also.

